# Unproven stem cell-based interventions & physicians’ professional obligations; a qualitative study with medical regulatory authorities in Canada

**DOI:** 10.1186/1472-6939-15-75

**Published:** 2014-10-14

**Authors:** Amy Zarzeczny, Marianne Clark

**Affiliations:** Johnson-Shoyama Graduate School of Public Policy, University of Regina, Regina, Canada; Health Law Institute, University of Alberta, Edmonton, Canada

**Keywords:** Stem cell tourism, Medical tourism, Professional regulation, Professional discipline, Policy, Ethics

## Abstract

**Background:**

The pursuit of unproven stem cell-based interventions (“stem cell tourism”) is an emerging issue that raises various concerns. Physicians play different roles in this market, many of which engage their legal, ethical and professional obligations. In Canada, physicians are members of a self-regulated profession and their professional regulatory bodies are responsible for regulating the practice of medicine and protecting the public interest. They also provide policy guidance to their members and discipline members for unprofessional conduct.

**Methods:**

We conducted semi-structured telephone interviews with representatives from six different provincial Colleges of Physicians and Surgeons in Canada to discuss their experiences and perspectives regarding stem cell tourism. Our focus was on exploring how different types of physician involvement in this market would be viewed by physicians’ professional regulatory bodies in Canada.

**Results:**

When considering physicians’ professional obligations, participants drew analogies between stem cell tourism and other areas of medical tourism as well as with some aspects of complementary alternative medicine where existing policies, codes of ethics and regulations provide some guidance. Canadian physicians are required to act in the best interests of their patients, respect patient autonomy, avoid conflicts of interest and pursue evidence-based practice in accordance with accepted standards of care. Physicians who provide unproven treatments falling outside the standard of care, not in the context of an approved research protocol, could be subject to professional discipline. Other types of problematic conduct include referrals involving financial conflict of interest and failure to provide urgent medically necessary care. Areas of ambiguity include physicians’ obligations when asked for information and advice about seeking unproven medical treatments, in terms of providing non-urgent follow-up care, and when asked to support efforts to go abroad by providing tests or procedures in advance that would not otherwise be medically indicated.

**Conclusions:**

Specific policy guidance regarding the identified areas of tension or ambiguity may prove helpful for physicians struggling with these issues. Further consideration of the complex interplay of factors at issue in how physicians may (should) respond to patient demands related to unproven medical interventions while meeting their professional, legal and ethical obligations, is warranted.

## Background

The practice of medicine is unquestionably complex and the challenges Canadian physicians and their counterparts around the world face on a daily basis are numerous and multi-faceted. Medical tourism – where patients deliberately travel to receive medical care outside of their home jurisdiction – is one such challenge. Medical tourism is far from a new phenomenon and comes in many forms. In Canada, as is true in other countries, some individuals travel outside their home jurisdiction to pursue a wide range of medical interventions, some of which are standard therapies (e.g. dental surgery, orthopedic surgery, etc.) that may simply be more accessible (either financially or in terms of wait times) in other jurisdictions. In other cases, people travel to pursue treatment options that are not available domestically for some reason such as legal barriers (e.g., commercial surrogacy) or insufficient evidence of safety and/or efficacy (e.g., liberation therapy to treat chronic cerebrospinal insufficiency venous syndrome (CCSVI)) [1].

One particular type of medical tourism receiving increasing attention is the pursuit of unproven stem cell-based interventions, often referred to as “stem cell tourism” [2]. Private clinics around the world are advertising unproven stem cell-based interventions on a direct-to-consumer basis over the internet. These treatments are offered for a wide range of conditions, including autism, ALS, spinal cord injury and multiple sclerosis, among many others [3–5]. Although evidence on the veracity of the claims made on clinic websites, on patient blogs and in newspaper reports is lacking, various cell sources are purportedly used including (but not limited to) adult autologous, fetal, umbilical cord and embryonic [6]. Patients typically pay for these treatments out-of-pocket and, although robust data is lacking, newspaper coverage reflects an average cost of approximately $47,315.00 USD [7]. The primary concern associated with this emerging market is risk to participating individuals. Reported adverse consequences include meningitis [8], tumours [9], other lesions [10], and death [11]. The long-term risks of currently unidentified or unanticipated adverse consequences also should not be underestimated.

Although we have adopted the term “stem cell tourism” for the purpose of this study because it has been widely used in previous research in the area, we do so in recognition of several important caveats. First, we recognize the concern that the use of “tourism” may be seen to minimize the incredibly difficult realities many individuals involved are dealing with [12], and in no way suggest these pursuits are undertaken lightly or without significant hardship. The term may also be misleading if it is taken to mean that travel is necessarily involved. In the early stages of the developing international market for unproven stem cell-based interventions, concerns tended to focus on patients travelling from Western countries to clinics operating in countries with less stringent regulatory regimes [3–5]. However, increasingly the debate is turning to more tightly regulated countries such as the United States and Australia and the emerging practice of autologous stem cell treatments [13]. These developments are serving to heighten debates surrounding physicians’ conduct in providing autologous stem cell-based interventions that have not been proven safe or effective by traditional means and makes the research presented here particularly timely. This emerging practice also highlights a key point for discourse and policy work addressing the stem cell tourism market – the location of the intervention is not of central importance; its unproven nature however *is*.

Physicians play various roles in the stem cell tourism market. For example, in some cases they provide unproven stem cell-based interventions directly to their patients [14, 15]; in other cases they advertise or offer referrals to providers in other jurisdictions [16]. In yet other situations they act as sources of information and advice for their patients [17, 18], among various other types and degrees of involvement (e.g., clinic ownership, membership on advisory boards, suppliers of cell lines, etc.). Even when physicians are not directly involved in providing the unproven interventions, research from other areas of medical tourism suggests that physicians are being asked by patients for advice and direction about procedures offered elsewhere [19], and are also being approached by patients seeking medical records or particular diagnostic tests to prepare for treatment abroad [20]. They may further be asked to provide follow-up care upon a patient’s return from receiving unproven treatment elsewhere – as was the case with Canadian multiple sclerosis patients returning from having had liberation therapy for CCSVI outside the country [21, 22]. Each of these circumstances raises different potential issues for the physician involved from ethical, legal and practical perspectives.

In Canada, as is true in other jurisdictions around the world, medicine is a self-regulated profession. All ten provinces in the country have a College of Physicians and Surgeons that is empowered by provincial legislation to regulate the practice of medicine in that province and the three territories operate on a similar basis (Table 1). The Colleges are typically responsible for licensing physicians, maintaining standards of practice, investigating complaints and disciplining members. They are also generally required to act in the public interest and/or to protect the public (e.g., *Medical Profession Act, 1981*, s. 69.1) [23]. Accordingly, these medical regulatory authorities have a vital role to play in the oversight of members’ conduct including in relation to their involvement in or experience with stem cell tourism and other forms of medical tourism. In this paper, we explore how medical regulatory authorities in Canada respond to the issues raised by these practices, with a view to clarifying physicians’ professional responsibilities in this and related contexts. The legal and professional obligations physicians have when encountering unproven stem cell-based interventions is a topic that has received some consideration [17], and which merits further enquiry given the complexities and concerns surrounding this emerging area of medical tourism.

**Table 1:** **Overview of medical self-regulation in Canada**

Province/Territory	Medical Regulatory Authority	Empowering Legislation
British Columbia	College of Physicians and Surgeons of British Columbia	*Health Professions Act*, R.S.B.C. 1996, c. 183
Alberta	College of Physicians and Surgeons of Alberta	*Health Professions Act*, R.S.A. 2000, c. H-7
Saskatchewan	College of Physicians and Surgeons of Saskatchewan	*The Medical Profession Act*, *1981,* S.S. 1980–81, c. M-10.1
Manitoba	College of Physicians and Surgeons of Manitoba	*The Medical Act*, C.C.S.M. 2009, c. M90
Ontario	College of Physicians and Surgeons of Ontario	*Regulated Health Professions Act*, S.O. 1991, c. 18
		*Medicine Act*, S.O. 1991, c. 30
Quebec	Collège des médecins du Quebec/College of Physicians and Surgeons of Quebec	*Medical Act*, R.S.Q. 1973, c. M-9
Nova Scotia	College of Physicians and Surgeons of Nova Scotia	*Medical Act*, S.N.S. 1995–6, c. 10
Prince Edward Island	College of Physicians and Surgeons of Prince Edward Island	*Medical Act*, RSPEI 1988, c. M-5
New Brunswick	College of Physicians and Surgeons of New Brunswick	*Medical Act*, S.N.B. 1981
Newfoundland and Labrador	College of Physicians and Surgeons of Newfoundland and Labrador	*Medical Act*, SNL 2011, M-4.02
Yukon	Yukon Medical Council	*Medical Profession Act*, R.S.Y, 2000, c. 149
Northwest Territories	Department of Health and Social Services	*Medical Profession Act*, S.N.W.T. 2010, c.6
Nunavut	Department of Health and Social Services	*Medical Profession Act*, R.S.N.W.T. 1988, c. M-9

## Methods

We conducted a qualitative study based on semi-structured interviews with representatives from Colleges of Physicians and Surgeons in Canada. All of our participants were in leadership and/or senior administrative positions with knowledge of both policy and disciplinary activities of their College. Some had medical backgrounds, but all spoke from the perspective of a representative of their College, as opposed to that of a practicing physician. The aim of these interviews was to gain insight into their experiences and perspectives in relation to stem cell tourism, in order to understand how different kinds of physician involvement in stem cell tourism might be dealt with by professional self-regulatory authorities in Canada. This research was approved in advance by the Research Ethics Boards at the University of Regina and the University of Alberta. All participants provided informed consent before taking part in this study.

### Recruitment

Letters of invitation were sent by email to the Registrar of each College of Physicians and Surgeons in Canada (and their counterparts in the territories), using contact information provided on their public websites. Invitations were accompanied by information about the research project and a consent form. Responses were received from eight Colleges. Two of those eight declined to participate, citing lack of familiarity and/or experience with the area, and six agreed. Recruitment efforts ceased after one round of follow-up emails sent to unresponsive invitees. Semi-structured interviews were conducted by telephone between September, 2013 and January, 2014. All interviews were conducted by the same investigator (AZ) to promote consistency.

### Data collection

A semi-structured interview approach [24] was adopted to ensure key areas of enquiry could be addressed while leaving considerable room and flexibility for participants to raise unanticipated issues and topics. In advance of the interviews, participants were provided with an introduction to stem cell tourism (Appendix A) as well as three short case studies (Appendix B), each based on an actual instance of physician involvement in stem cell tourism. The cases were used to provide examples and context to the questions posed during the interviews.

The interviews canvassed a variety of different areas including physician referrals to treatment providers located in other jurisdictions, physicians actually providing unproven interventions, physicians providing follow-up care to patients upon their return from receiving treatment elsewhere, physicians providing information and advice to patients asking about stem cell-based interventions available in other jurisdictions, and reach of the regulatory jurisdiction or authority of the Colleges over their members. Broadly, we were interested in learning whether the Colleges had any experience with stem cell tourism to-date and, even if they had not, how they anticipated different kinds of situations would be viewed or dealt with under their existing regulatory regime. To that end, different examples (emerging from the case studies) were put to the participants and they were asked for their reactions, including how they would anticipate such a case being dealt with if it involved a doctor licensed by their College.

### Analysis

Interviews generally ran from between thirty to sixty minutes. They were digitally recorded and later transcribed verbatim. Both investigators reviewed all transcripts and notes independently to identify emerging themes relevant to the overarching research objectives. These themes were compared and discussed by both investigators and a preliminary coding scheme that captured the major topics in the data was developed [24]. This coding scheme consisted of a ‘long list’ of initial themes.

The second author then coded the data by placing relevant phrases and sentences from the transcripts into these thematic categories. The coded data was subject to further review and iterative analysis by both authors. Authors engaged in frequent conversation to further refine and interpret these themes. Ultimately, five overarching themes were agreed upon that addressed the research objectives and captured ideas identified deductively in the transcripts [25].

## Results

A number of clear themes emerged. There were interesting points of agreement upon which participants were fairly consistent in their conclusions regarding the utility of existing guiding principles. There were also a number of areas where the discussions reflected various ambiguities and tensions.

### Drawing on existing frameworks

Although only one participant had dealt directly with stem cell tourism, all participants recognized familiar issues raised by this practice. In anticipating how their College might respond to different kinds of circumstances in the stem cell tourism arena, including those presented in the case studies, many drew analogies with other topics with which they were more familiar. These analogies often related to other areas of medical tourism and, in a couple of cases, complementary alternative medicine.

Participants generally agreed that both medical tourism and complementary alternative medicine - and, by extension, stem cell tourism - raise a number of issues from a regulatory perspective, some of which can be addressed in a fairly straightforward manner under established principles (discussed more below), while others present greater challenges. Almost all participants made specific reference to the controversies that emerged surrounding CCSVI and drew analogies between the challenges Canadian physicians and Colleges experienced in relation to that phenomenon, and what might be anticipated to occur in the context of stem cell tourism. Although medical tourism was not found to be an exceptionally persistent concern for Colleges in Canada at the moment, participants recognized the potential for increasingly complicated scenarios to emerge as it becomes more commonplace.

### Where the lines are (somewhat) clear: conflict of interest, the rules for research and evidence-based practice

There was consensus among participants that conflicts of interest are a source of significant concern for the Colleges. For example, participants were in agreement that referrals to clinics or treatment providers with which a physician has a financial relationship (e.g., offshore clinics owned in whole or part by the Canadian physician or from which the Canadian physician receives a referral fee) would in most cases constitute an unacceptable conflict of interest. As one participant noted: *“Physicians … are precluded from getting any financial benefit from [referral] activity”* (P1). Another elaborated: *“The physician’s interest should never be put before patient interest…there shouldn’t be a financial benefit strictly for a referral. Referrals are an important part of care”* (P2). Another stated*: “Well I think it is all kind of shades of grey but the worst is when the physician is part of the ownership of the clinic. That would be the most egregious and you know, just because to do that work in Canada would be illegal, unethical and unprofessional doesn’t make it right if you then refer people [for that same treatment] outside of our jurisdiction. So we would take a dim view of that”* (P3).

Participants also agreed that when looking at treatment provided by a physician, a central question from a regulatory perspective is whether the treatment meets the standard of care and has an appropriate evidentiary foundation. As one participant noted, “*generally speaking a very key foundational element is that physicians shouldn’t be doing things that are not approved*” (P2). For example, as noted above some physicians in jurisdictions such as the United States are apparently providing adult autologous stem cell treatments for orthopedic injuries (potentially among other indications). A debate has emerged in the United States regarding whether this practice should or should not fall under the regulatory authority of the FDA [26, 27] and whether it may in fact violate physicians’ professional and ethical duties [28].

Our participants generally expressed that if an intervention provided falls outside the approved standard of care and in the absence of proof of efficacy, then it must be determined whether the physician is engaging in research. If so, participants identified existing research ethics and clinical trial approval processes which govern research behaviour and expressed that physicians should not engage in research involving patients outside of these frameworks. In some cases, clinical research may present options for individuals who have encountered the limits of conventional treatment. However, human subjects research is subject to and guided by clearly defined rules and processes, including requirements for informed consent and an appropriate risk-benefit assessment. Participants agreed that any physician referring or providing experimental treatment or unproven procedures would generally be required to do so in the context of approved clinical research. As one physician stated: “*Non-standard therapy can only be provided when it is part of an appropriately constituted research ethics board approved trial*” (P3).

Some participants also noted the importance of informed consent in this context – i.e. when treatment is proposed that falls outside conventional therapy – and reflected on the importance of a robust risk/benefit analysis. Participants did not engage in detailed discussion of medical innovation in this context, beyond comments recognizing the speed with which emerging fields of science can move – discussed further below.

### Managing expectations: where clarity erodes

The utility of existing regulatory frameworks to guide the conduct of physicians only extended so far. Participants described several examples of physicians’ indirect involvement in medical tourism generally, or stem cell tourism in particular, where the line between appropriate and inappropriate conduct was less clear. These examples often related in one way or another to the management of patients’ expectations.

One area several participants struggled with (or differed from one another on) was the extent to which physicians are or are not obligated to be (or become) informed about novel or unproven interventions. One scenario familiar to several participants was that of having a patient ask his/her doctor for additional information or advice regarding a treatment he/she had heard about elsewhere – often via information obtained on the internet. For example, one participant shared the following thoughts:

“*In years past they didn’t have the Internet, they would have some access, support groups and a few other things, but they wouldn’t have 500 sheets that they just printed off this morning which they want you to read, and doctors do express some frustration with that… There is nothing that comes back without a search in Google but patients are not really in a position to separate the wheat from the chaff*” (P1).

Another participant explained the College’s process in responding to related queries:

“*We do sometimes get inquiries from physicians to say well what kind of role do I play if my patient tells me that they want to go out of province or out of country or something and our normal advice is, well, you provide them the same guidance you provide them any other time. If you have an opinion with respect to the medical decision that they are going to undertake, then you try to provide as much balanced information as possible so that they can make the best decision for themselves*” (P4).

There was general recognition of the challenges physicians face in these contexts and the very real limitations that exist in terms of physicians’ knowledge, expertise and available time. In other words, participants who discussed this issue were fairly united in the view that physicians simply cannot be expected to know everything about all cutting-edge research possibilities or about every therapy/intervention available on the private market – particularly in other countries. As one participant stated: “*I don’t think I would expect a physician to be fully aware of what happens in Costa Rica. I mean there is a limit as to the expectations that a physician should be knowledgeable about*” (P3).

Participants differed somewhat in terms of how far they would expect a physician to go to become informed. Some expressed that it would be fine for a physician to simply say he/she did not know: “*Physicians must tell patients what information they have. If they know something about something they should mention it. They have a right to inform their patients. They would also be responsible for informing patients if they don’t know what it is all about and if they are dubious or don’t have any information on whether it is a safe or successful therapy*” (P5). Others felt physicians may have some obligation to at least refer the patient to a more knowledgeable source of information. For example, one participant suggested that in some circumstances,

*“I think they would perhaps be expected to pick up the phone and particularly a family doctor who has got an ongoing continuity of care relationship with a patient who kind of co-manages them with say a cancer agency or the MS clinic or an orthopedic surgeon, to at least discuss it with the consulting doctor so they have that doctor to doctor conversation; but it may be ‘I am sorry I have looked into this. This is not standard therapy; I can’t really assist you in making an informed choice about this because it is beyond what we do’*” (P3).

In the words of another participant: “*they should consider whether they can assist patients in obtaining information and it might involve suggesting resources to the patients or referring patients to other practitioners if it is in the patients’ best interest and will support their position. So there is no strong obligation to know about things. It is more of think about whether you can help your patient*” (P2). Some participants distinguished between specialists and general practitioners, to the extent that the onus may be stronger on specialists to be abreast of emerging developments (including current research) in their field.

Another issue participants identified as being potentially problematic related to physicians being asked by patients to provide pre-procedure testing and/or post-treatment follow-up care. Indeed, this was the area some participants identified as having had the most experience with in the medical tourism context. One participant expressed the following: “*The majority of the calls we get are more related to when something goes wrong and these patients have come back into the system - what responsibility do physicians, say in the emergency departments or some specialists like plastic surgeons, have when they are called to see a patient who has received treatment from a treating physician outside the country*?” (P4).

A number of participants identified these situations as having emerged as areas of concern in the context of the CCSVI phenomenon in Canada. When it came to the question of pre- procedure testing, a central question from a professional regulatory perspective was whether the test would have been ordered in any event. One participant suggested the College would provide the following advice to a physician wondering how to deal with a request for pre-procedure testing: “*if you don’t think this is useful then you are under no obligation to do so and we would question why you would do so*” (P6).

In terms of providing post-treatment care, participants often distinguished between emergent care needs and procedure-specific follow-up. An example given was when Canadian physicians were asked to provide follow-up ultrasounds for patients who received liberation therapy to treat CCSVI out of the country. These situations can be contrasted to when patients who received the same therapy experienced blood clots or other medical crises upon return. One participant provided the following context:

*“We needed to remind people that just because they went and got an experimental procedure that the doctor didn’t agree with didn’t mean that the doctor could provide no care. They were certainly not ethically obliged to provide follow-up imaging or repeat the procedure which had been done somewhere else, but they did need to be alive to urgent emergent situations that might be a consequence of that therapy, such as a stent that had moved, or infection or a complete blood clot or something like that obviously would require urgent emergent care*” (P3).

Participants generally agreed that overall, physicians were obliged to provide emergent care in accordance with existing standards of care and regulatory frameworks. One suggested the following: *“the code of ethics addresses the circumstances for which you can provide care to a patient, [and] the circumstances in which you could deny or refuse to provide care to a patient*” (P4). Others echoed this sentiment and suggested that physicians are ethically required to treat patients regardless of whether they agree with their decisions (i.e. to pursue treatment elsewhere) or not. One stressed that physicians should not base their care decisions on an ethical or valued appraisal of a patient’s decision. Having said that, a number of participants also acknowledged the difficulties their members encounter when asked to provide follow-up care for a procedure that falls outside the current standard of care, often in the absence of adequate information, and when the requested intervention would not otherwise be medically indicated.

### The physician-patient relationship: a balancing act

The idea that physician conduct should be guided by the patient’s best interests was stressed repeatedly by participants. However, there was also widespread recognition that in practice, determining the appropriate course of action in situations where patients want to pursue an unproven treatment, potentially outside their home jurisdiction, can be a challenge. Participants explained that physicians are often trying to balance respect for patient autonomy, the need and desire to act in their patients’ best interests, and the duty to meet the standard of care – and, in cases of medical tourism or complementary alternative medicine, often with imperfect information.

As one participant noted,

*“[Physicians] understand the desperation the patient feels. I mean the basic rules are you always have to consider the wellbeing of the patient; you should never do anything that you don’t think is in the patient’s best interests. But, at the same time you have to respect the patient’s right to accept or reject any medical treatment and, as I said, there has also been a long standing rule that they are to respect the patient’s request for another opinion from a physician of their choice*”(P1).

Discussions of patient autonomy were often linked to the importance of informed consent. For example, one participant noted the following:

“*we have a regulatory framework; we are looking at this from the perspective of the patient and that sort of push and pull between a patient being entitled to make an autonomous decision even if it is one that may not in the eyes of other people look like a good one. Patients are entitled to do so but fundamental to that is - does this patient know what he or she is fighting, have they been provided with enough information to know what they are getting into, how has the doctor represented the treatment?*” (P2).

Of course, one of the recognized challenges when an individual pursues care elsewhere is that his/her doctor in Canada will often have few to no details regarding (and certainly no control over) what information the individual may or may not have received about the particular intervention and its attendant risks.

A related tension acknowledged in various ways by different participants was the concern physicians may feel about damaging the doctor-patient relationship such that future care may be compromised. For example, one participant reflected on experience with physicians who have been asked for an opinion about an unproven treatment and who fear that providing a critical perspective may not dissuade the patient but rather make them reluctant to return to him/her for necessary care afterwards, thereby damaging continuity of care (and perhaps exposing the patient to additional risk). “*It would probably be more a situation of discussion and advice as opposed to saying ‘no you cannot do this’ because in some cases the physician worries about affecting the doctor/patient relationship by refusing to order the test and so they make a decision that overall it is not a bad thing*” (P6). Another participant noted: “*certainly the trusting relationship between the doctors and patients is something we are very, very much alive to in terms of the policy work that we do*” (P2). Indeed, the importance of the doctor-patient relationship emerged at various points throughout the interviews with recognition given to the concern physicians may feel about damaging it.

### Moving forward: challenges and opportunities

While participants acknowledged various challenges their members currently face in relation to different aspects of medical tourism, it seems unlikely we will see any significant policy focus on this area any time soon, absent a fairly dramatic rise in associated complaints or calls for guidance from physicians. The general approach to addressing medical tourism from a regulatory perspective in Canada is currently more reactive than proactive, perhaps because the Colleges are not encountering issues and controversies related to medical tourism frequently or dramatically enough to merit action. As one participant put it: “*We can’t be everything to all people all the time and so you tend to focus your policy initiatives in those areas where there appears to be an issue of concern*” (P6). Participants also indicated that regulation should be guided by the desire to ensure quality of care and not necessarily to ‘police’ conduct; in some cases we heard a mild caution not to engage in regulation for the sake of regulation. Another participant expressed: “*there is a limit to what we can do as regulators*” (P3).

In addition to these practical and principle-based factors, participants also identified a number of challenges that may preclude or deter proactive efforts in the area of stem cell tourism. The ever changing and expanding boundaries of globalized health care were one such factor; another related to the limits not only of physicians’ knowledge as individuals, but the knowledge of the field, in that there is a continuously shifting and precarious line drawn between standard of care and innovation, between evidence and hope. As one participant said, “*what might be experimental treatment now could be standard care in two years*” (P3). This line of thought seems particularly relevant in the context of the debates referenced earlier regarding the administration of autologous stem cell-based interventions.

Interestingly, the long reach of the regulatory arm emerged as a significant strength in the ability of professional regulation to oversee and address problematic conduct on the part of physicians, as did its focus on protecting the interests of patients and the public. Although participants varied somewhat in their levels of conviction, all acknowledged that their College would certainly take an interest in the conduct of their members even where it took place outside their jurisdiction. Some went on to confirm that their physicians are subject to their rules, policies, and disciplinary processes, wherever they provide care. And all were very much alive to the responsibility self-regulated professions have to protect the public interest.

## Discussion

As members of a self-regulated profession, Canadian physicians are governed and guided by their respective professional regulatory body which, at the provincial level, is the relevant College of Physicians and Surgeons. As outlined above, in this research we interviewed representatives from six provincial Colleges of Physicians and Surgeons in Canada to investigate their perspectives on stem cell tourism and how different types of physician involvement would be viewed by the College. A number of themes emerged from these discussions, centering on both areas of clarity and ambiguity, and many of these echo previous research on related areas of medical tourism. It was perhaps no surprise that in general our participants expressed that many of the issues raised by stem cell tourism are not specific to this area and that there are existing guidelines, regulations and policies that would apply to some of the challenges the area shares with analogous issues [29–31] .

Nonetheless, it is also apparent that guidelines, regulations and policy can only go so far and the specific facts at hand in a particular case are always of central importance. The nature of the doctor-patient relationship at issue, the apparent motivations of the physician and the specific circumstances of the patient all play a role in how complaints are evaluated. While this reality is arguably one of the benefits of the professional disciplinary process, it must also be noted that it carries some challenge for meaningful prospective policy guidance. As one of our participants noted a number of times throughout our discussion, many of these questions and their answers fall in “shades of grey”. Indeed, the complexities and nuances engaged in determining what may or may not be unprofessional conduct on the part of physicians in the types of circumstances addressed in this research should not be underestimated. Unfortunately, this reality may leave a number of important questions unanswered when it comes to providing support for busy physicians seeking to respond in real-time to the needs and requests of their patients.

It was clear from all participants that Canadian physicians are required to act in the best interests of their patients, to respect patient autonomy, ensure appropriately informed consent, avoid conflicts of interest and pursue evidence-based practice in accordance with accepted standards of care. Although these basic principles are laudable and seemingly straightforward, it remains to be seen how they will be applied and interpreted if and when the provision of unproven stem cell-based interventions comes closer to home. To-date, we in Canada are not yet seeing the same rise in adult autologous stem cell transfers as are our American neighbours. When asked about this practice, the majority of our participants turned their focus to two key questions – first, whether there is evidence to support the treatment, and second, if not, whether it is being provided in the context of an approved research protocol. As noted above, some also highlighted the importance of informed consent in this context, the role of evidence and scientific rationale for treatment protocols, and of ensuring an appropriate risk/benefit ratio. However, it is yet to be determined how the Colleges would respond if Canadian physicians were to enter this market by routinely providing autologous stem cell-based interventions. It also remains to be seen how this practice would relate to law and policy surrounding medical innovation, compassionate access and ‘right to try’ movements, among other issues. It is beyond the scope of this paper to engage in such an analysis here, but further study on these questions is merited.

Other types of conduct Canadian Colleges of Physicians and Surgeons are likely to take a dim view of include referrals where a physician has a financial interest and failure to provide urgent medically necessary care, regardless of the reason for the immediate medical need. It is also evident there are areas of tension that are not answered decisively by current regimes and where Canadian physicians’ obligations are less clear from a regulatory perspective. These areas of greater ambiguity relate primarily to less direct forms of physician involvement in stem cell tourism, including how far physicians are required to go when asked for information and advice about seeking medical treatment (including a stem cell-based intervention) outside the jurisdiction, what their obligations are in terms of providing non-urgent follow-up care, and to what extent they can/should/should not support efforts to pursue care elsewhere by providing tests or procedures that would not otherwise be medically indicated.

These tensions have been identified in other research on medical tourism [32, 19], along with concerns that physicians may fear negative impacts on their relationships with their patients (which may threaten continuity of care) if they refuse requests for pre-procedure testing [19], and questions regarding the implications of having minor patients – or others lacking decision-making capacity – involved [33]. These concerns, echoed by a number of our participants, are also areas that arguably have the greatest implications for the health care system as a whole, in that they involve the expenditure of public health care dollars and engage the role of the state in supporting and at times protecting the health of its citizens. The empirical data presented in this research supports previously identified needs for greater clarity and guidance to be made available to physicians who are grappling with these issues in their daily practices.

### Limitations

We did not interview representatives from every provincial or territorial medical regulatory body in Canada so these data cannot be considered comprehensive. The interviews reflect the perspectives of individual participants in leadership roles but also cannot necessarily be interpreted as reflecting the views of everyone involved in the regulatory process or in a given College. Nonetheless, these results present useful insight into how different kinds of physician involvement in the stem cell tourism market would likely be viewed by medical regulatory authorities in Canada. They also point to areas where future policy development may be most important. Given similarities in general principles of professional regulation, these insights may also be broadly applicable to other jurisdictions with comparable professional regulatory regimes for physicians.

## Conclusions

It is the areas of tension or ambiguity identified in this research where future policy work in the form of guidance from medical regulators to their members may ultimately prove particularly useful. As professional self-regulatory bodies, Canadian Colleges of Physicians and Surgeons have significant power and authority over the medical profession. They also are a tremendous resource to their members when it comes to providing advice and guidance in relation to challenging areas of practice. In the absence of specific guidance produced by their College, these data indicate that at present Canadian physicians should look to existing codes of ethics, standards of practice, regulations and policies to guide their conduct in circumstances where they may feel conflicted or challenged by issues associated with stem cell tourism and arguably, other areas of medical tourism.

Future research examining the extent to which physicians are aware of and act in accordance with these frameworks would be valuable. If medical tourism in general continues to grow in Canada, and if stem cell tourism in particular becomes an issue more Canadian physicians are faced with on a routine basis, the Colleges may be called upon to provide more direct advice or guidance to their members regarding how to deal with the areas of tension identified above. Should that occur, it appears from these interviews that they will be well-placed to do so given the wealth of existing principles and guidance they have to drawn upon. These are important issues for patients, healthcare providers and health policy leaders, and as global health markets continue to develop it is essential these conversations continue.

## Appendix A

### Background information provided to participants

Stem cell tourism is the phrase commonly used to describe the phenomenon whereby individuals pursue stem cell-based interventions that have not been proven to be safe, effective or ready for routine clinical use. Clinics around the world offer these stem cell-based interventions to treat innumerable conditions ranging from autism to ALS, heart disease to spinal cord injury, multiple sclerosis to aging, often at great expense to the individual patient. These services are generally marketed online, on a direct-to-consumer basis. In many cases, patients travel (hence the inclusion of “tourism” in the description) to pursue these interventions in jurisdictions that historically have had less stringent regulation in this area (e.g., China, India and Mexico). However, increasingly these transactions are also being reported in jurisdictions with well-established regulatory controls for scientific research and development, such as the U.S. Concerns associated with stem cell tourism are numerous and include risks to participants’ health, questions regarding the accuracy of the information communicated to prospective patients (i.e. how the treatments are portrayed and the degree to which risks and unknowns are explained), the financial strain associated with high treatment costs and travel expenses and threats to the legitimacy of and long-term support for the stem cell research field.

## Appendix B

### Case studies presented to participants

#### Case Study A: Dr. T, UK

Dr. T was brought before the United Kingdom’s General Medical Council (GMC) to assess his fitness to practise, following accusations of misconduct with regard to stem cell-based treatments for multiple sclerosis he provided both in England and in the Netherlands. In a number of instances, he met with the patient first in London but subsequently provided treatment at his clinic in the Netherlands. The Fitness to Practice Panel determined that Dr. T’s fitness to practise was impaired by reason of his misconduct. Dr. T’s actions constituted “repeated and serious breaches of many of the essential tenets of ‘Good Medical Practice’”. The Panel’s findings included the following: Dr. T failed to respect the rights of the patient to be fully informed; he abused his position as a doctor; his treatment lacked the rigour required for a medical practitioner to embark on pioneering treatment, including lack of physical assessment pre-and post-treatment, lack of expertise in the research area and lack of follow-up consultation or care. The Panel directed Dr. T’s name be erased from the Medical Register, noting that UK physicians are bound by the standards required by the GMC despite carrying out their duties outside of this jurisdiction.

#### Case Study B: Dr. W, Singapore

Dr. W was registered as an obstetrics and gynaecology physician in Singapore when he was convicted of professional misconduct for advertising a service in which he would provide pre-and post-treatment care and accompany patients to foreign clinics where they would receive stem cell therapy for facial and body rejuvenation. The disciplinary committee found the location of these treatments was irrelevant and he should have known the treatments were not medically proven and therefore prohibited. The resulting sanction included a $10,000 fine (in addition to the costs of the hearing), censure, and a written agreement to refrain from these activities.

#### Case Study C: Dr. P, USA

Dr. P was an orthopedic surgeon who injected his patient with autologous stem cells (fat and bone marrow cells) to treat ligament damage to his elbow and shoulder. The patient reportedly recovered from the injuries and was very satisfied with the treatment provided.
